# Hexameric-Based Hierarchy in the Sizes of a Cytolysin Pore-Forming Complex

**DOI:** 10.3390/biom15030424

**Published:** 2025-03-17

**Authors:** Meijun Liu, Xintao Qin, Menglin Luo, Yi Shen, Jiabin Wang, Jielin Sun, Daniel M. Czajkowsky, Zhifeng Shao

**Affiliations:** 1 State Key Laboratory of Systems Medicine for Cancer, School of Biomedical Engineering, Shanghai Jiao Tong University, Shanghai 200240, China; mj.liu@sjtu.edu.cn (M.L.); a843261090@sjtu.edu.cn (X.Q.); luomenglin@sjtu.edu.cn (M.L.); shenyi12@sjtu.edu.cn (Y.S.); zfshao@sjtu.edu.cn (Z.S.); 2 Xiangfu Laboratory, Jiashan 314100, China; wjb0221@sjtu.edu.cn; 3 Institute of Translational Medicine, Shanghai Jiao Tong University, Shanghai 200240, China; jlsun@sjtu.edu.cn

**Keywords:** cholesterol-dependent cytolysins, pore-forming toxins, multi-stack gel electrophoresis, allostery, structural coordination, atomic force microscopy

## Abstract

Perfringolysin O (PFO) is a prototypical member of a large family of pore-forming toxins (PFTs) that are potent virulence factors for many pathogenic bacteria. One of the most enigmatic properties of these PFTs is how structural changes are coordinated between different subunits within a single complex. Moreover, there are conflicting data in the literature, with gel electrophoresis results apparently showing that pores are only complete rings, whereas microscopy images clearly also show incomplete-ring pores. Here, we developed a novel multi-stack gel electrophoretic assay to finely separate PFO pore complexes and found that this assay indeed resolves both complete- and incomplete-ring pores. However, unexpectedly, we found that the stoichiometries of these complexes are predominantly integral multiples of six subunits. High-resolution atomic force microscopy images of PFO pore complexes also reveal a predominant hexameric-based stoichiometry. We also observed this hexameric-based stoichiometry at the prepore stage and identified a mutant that is kinetically trapped at a hexameric state. Thus, overall, these results reveal a previously unknown hexameric-based structural hierarchy in the PFO complexes. We suggest that the structural coordination within the hexamers is different than between the hexamers and is thus a critical feature of the structural coordination of the complex as a whole.

## 1. Introduction

Cholesterol-dependent cytolysins (CDCs) are a large family of bacterial pore-forming toxins (PFTs) that play significant roles in virulence and are part of a larger family of membrane attack complex/perforin-like proteins (MACPF)/CDCs that function in both virulence and immunity [1,2,3,4,5,6,7]. The CDCs bind to the bilayer as monomers, self-assemble into prepore intermediates on the bilayer surface, and then cooperatively insert into the bilayer to form membrane-spanning pore complexes [8,9]. One of the properties that is unique among the CDCs when compared to the smaller bacterial PFTs is that they form a very wide range of sizes, including those that are very large, containing many tens of subunits [10,11,12,13]. Thus, beyond the interest in studying the pathological properties of these proteins, there is also interest in studying these proteins as model systems of large biological complexes [3]. In particular, a long-standing question of these PFTs is how the structural changes within each subunit are coordinated among the different subunits within a complex during the pore-forming process. Some other large biological complexes have been proposed to function via a so-called “nested hierarchical allostery” [14], in which subunits coordinate structural changes within sub-complexes of the whole complex and then there is coordination between these sub-complexes, but the generality of this mechanism is still unknown [15].

An early suggestion for the coordination within the CDCs was that all of the subunits within a single complex are, essentially, completely coordinated with each other [16]. That is, all subunits undergo the same change in structure during the prepore-to-pore transition simultaneously (cooperatively), triggered by the formation of a complete-ring-shaped complex [16]. With this idea, all pore-forming complexes must necessarily be complete-ring complexes. Consistent with this, previous studies have shown that sodium dodecyl sulfate-agarose gel electrophoresis (SDS-AGE) of CDC complexes yields only a single predominant oligomeric complex [16,17,18,19,20,21,22]. However, images of pore complexes in lipid bilayers obtained by atomic force microscopy (AFM), electron tomography, and negative-stain electron microscopy have clearly shown heterogeneously sized arc-shaped complexes that are prominently present among complete rings [23,24,25,26,27,28]. Clearly, such a wide range of sizes would be expected to produce many different bands (perhaps as many as tens of bands, one for each possible size of the pore complex) or a smear in electrophoresis gels (depending on the resolving power of the gels) and, thus, these observations are in apparent disagreement with the aforementioned gel results. Moreover, the presence of arc-shaped pore complexes would indicate that the completion of a ring complex is not the trigger for the coordinated structural change. Recent real-time single-molecule fluorescence microscopy data of the pore-forming process of perfringolysin O (PFO), a well-studied member of the CDC family, provided additional support that incomplete rings can indeed form pores [29,30]. Interestingly, this recent work also revealed that some complexes appear to continue to grow in size after the pore formation, in apparent conflict with the aforementioned prepore model. Hence, resolution of this issue is expected to not only provide clarity regarding this apparently disparate data but also reveal insights into the mechanism of pore formation.

Here, we re-investigated the sizes of the pore complexes of PFO by gel electrophoresis using a novel multi-stack polyacrylamide gel and indeed observed both complete- and incomplete-ring complexes of a range of sizes for the pore complexes. However, unexpectedly, rather than a large number of bands or a smear, there were generally only at most seven bands. AFM images of complexes isolated from these bands revealed that their contour lengths are integral multiples of six subunits. Moreover, AFM images of PFO pore complexes in supported lipid bilayers also revealed a predominant hexameric-based stoichiometry. We also show that prepore complexes are also predominantly hexameric and identify a mutant that is kinetically trapped at a hexameric state at room temperature. Thus, overall, these results point to an unexpected hierarchy in the structure of PFO complexes in which larger complexes are essentially composed of multiples of hexameric sub-complexes. We propose that different inter-subunit interactions within/between the hexamers result in a difference in the coordination of the prepore-to-pore transition within/between the hexamers, and thus these complexes function according to a nested hierarchy allostery, as observed with other large biological complexes.

## 2. Materials and Methods

### 2.1. Expression and Purification of PFO and Its Derivative

The sequences for the N-terminal His-tagged wild-type PFO, the disulfide-locked mutant PFO^G57C/S190C^, and the single-point mutant PFO^W165T^ were inserted into the pPROEXHTα vector by Sangon Biotech, and the same protein purification procedure was applied to each PFO as follows. The *E.coli* BL21(DE3) cells (TransGen Biotech, Beijing, China) containing recombinant plasmid were grown in LB medium with 0.01% ampicillin at 37 °C overnight, transferred into LB medium (1:100), grown until attaining an OD value of 0.8, and then induced with 1 mM isopropyl β-D-1-thiogalactopyranoside (IPTG, Sangon Biotech, Shanghai, China) at 16 °C for 18 h. Cells were harvested at 6000 rpm for 5 min, resuspended in PBS, and frozen–thawed for 3 cycles, after which the lysis buffer (0.15 mL CelLytic B (Sigma-Aldrich, St. Louis, MO, USA), 0.0015 g Lysozyme, 15 μL PMSF, and 0.5 μL Benzonase Nuclease (Yeasen, Shanghai, China) in 1.33 mL PBS) per 1 g wet cell pellet was added. The supernatant was incubated with ProteinIso Ni-NTA agarose Resin (TransGen Biotech, Beijing, China) (2000:1) rotating at 4 °C for 2 h. The resin was then washed thoroughly with 20 mM imidazole in PBS and then eluted with 300 mM imidazole in PBS. The protein solution was dialyzed and stored with 20% glycerol at −80 °C. The purities of all PFO complexes were evaluated with SDS-PAGE, and the hemolytic activity of the wild-type PFO was evaluated. For the latter, in short, the PFO was incubated with about 1 × 10^9^ erythrocytes from washed rabbit blood at 37 °C for 30 min. The percentage of hemolysis was determined by the release of hemoglobin monitored by the absorbance at 540 nm. The amount of toxin that produced 50% hemolysis was similar to published values [31].

### 2.2. Vesicle Preparation

Cholesterol, egg L-α-phosphatidylcholine (eggPC), and heart total lipid extract were purchased from Avanti Polar Lipids (Alabaster, AL, USA). The lipid powder was dissolved in chloroform to a stock concentration of 25 mg/mL. The solvent mixture was evaporated using nitrogen gas to form a homogenous dry lipid film. The lipid film was resuspended in 150 mM NaCl, 20 mM Hepes (pH 7.4) to a final concentration of 1 mg/mL and then sonicated to form unilamellar vesicles.

### 2.3. Native Gradient PAGE

**Figure 1:**
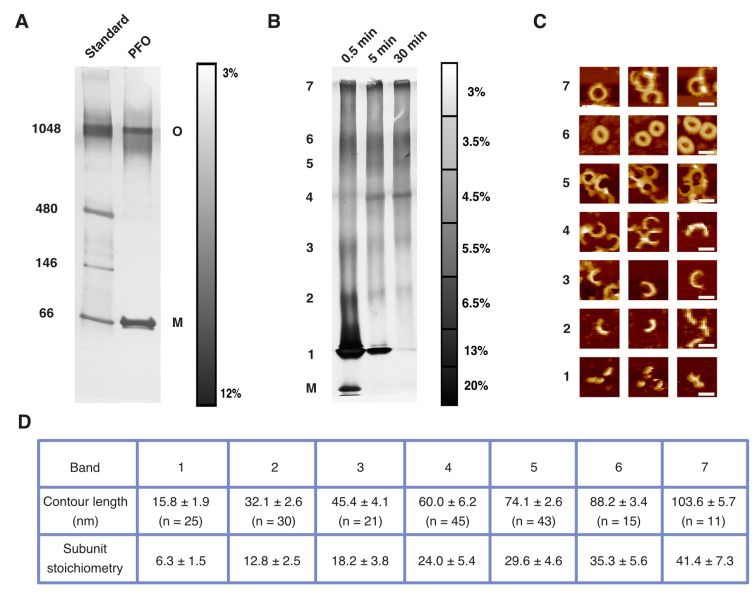
Gradient gel electrophoresis of PFO pore complexes. (**A**) A continuous 3–12% polyacrylamide gradient gel showing a predominant oligomeric band of PFO pore complexes in addition to the monomeric band (near 66 kDa). The scale bar reflects the gel density, and the numbers indicate molecular weights of the standard (left lane). The “O” and “M” labeled on the right side of the gel refer to oligomer and monomer, respectively. (**B**) The multi-stack gel reveals maximally seven distinct bands of pore complexes. The density of the stacks in the scale bar is determined by the volume of each stack that was cast. The times indicated above the gel refer to the incubation time of the PFO with the vesicles before solubilization with the loading buffer. (**C**) Typical AFM images of the complexes isolated from each of the individual bands (as numbered on the left in (**B**)). The complexes from the smaller five bands were predominantly incomplete rings, while the complexes in the larger two bands were mostly rings, with a minor portion of incomplete rings. Scale bar: 30 nm. (**D**) Measurements of the contour lengths from the individual bands. The lower row of subunit stoichiometry was obtained by dividing the contour length by the circumferential length of a single subunit (2.5 nm).

Continuous native gradient polyacrylamide gel electrophoresis (native gradient PAGE) is a common method for separating large protein complexes and is believed to afford a finer separation of differently sized complexes than agarose [32,33]. The 3–12% native gradient gel in Figure 1A was made using an automatic gradient former, and the NativePAGE 3–12% Bis-Tris gel in Supplementary Figure S1A was purchased from Invitrogen (Carlsbad, CA, USA). PFO (0.2 μL of 1 mg/mL) and the vesicle solution (20 μL of 0.1 mg/mL) containing eggPC–cholesterol 1:1 (molar ratio) were incubated at 37 °C for 30 min followed by the addition of the NuPAGE LDS 4× sample buffer (Invitrogen, Carlsbad, CA, USA). The solubilized complexes and the NativeMark unstained protein standard (Invitrogen, Carlsbad, CA, USA) or color prestained protein standard (NEB, Ipswich, MA, USA) were then loaded into the gel. Running conditions were 150 V for 60 min and 250 V for 30 min in a XCell SureLock Mini Cell (Invitrogen, Carlsbad, CA, USA) using NuPAGE Tris–Acetate SDS running buffer, pH 8.25 (Invitrogen, Carlsbad, CA, USA). Gels were stained by silver stain.

### 2.4. Multi-Stack Gel Electrophoresis

The gels were cast manually, stack by stack, with a minimum difference in cast gel density of 0.5% and a volume of 0.4 mL between neighboring stacks. Overall, we examined stacks with gel densities between 3% and 20%. The reagents used were free of reducing agents, and the samples were not boiled. A stock polyacrylamide solution of 40%T (T, weight percentage of the acrylamide) and 4%C (C, weight percentage of crosslinker) was pre-cooled, and APS and TEMED were both added to, at most, 0.1% (*v*/*v*). The casting process was finished in 2 min using a wide pipette tip. The sample preparation procedure of PFO and its derivative was the same as described above. A tris-tricine running buffer system (50 mM Tris, 50 mM Tricine, pH 8.3) was used. The gels were run by applying 26 V for 30 min, 50 V for 2 h, and then 70 V for 10 h to maintain a maximum current of 0.1 A. Gel staining was the same as described above for the native gradient PAGE. Multi-stack gel original images can be found in Supplementary Materials.

### 2.5. Gel Isolation and Imaging

One lane of the gel containing the sample was removed and stained to identify the bands. This lane was then put back into the gel and used as a marker to identify the appropriate positions within a lane of sample that was not stained. Each plug containing a single band was then placed into 2 mL centrifuge tubes and was ground manually and then placed in 1.5 mL water. The sample was then shaken for two days at 9 °C and concentrated by a Merck Amicon ultra-centrifugal filter (Sigma-Aldrich, St. Louis, MO, USA) with a 100 kDa pore size prior to AFM imaging. For AFM, the sample was applied to freshly cleaved mica and incubated for 30 min, followed by washing thoroughly with 150 mM NaCl, 20 mM Hepes, and 40 mM CaCl_2_ (pH 7.4). The contour lengths were measured at the centers of the arc–ring complexes. To account for the effect of tip broadening on this measurement, we estimated the magnitude of the tip-broadening effect based on the (radial) topographical profile within the middle of the complexes. We then subtracted this distance from each end of the contour to obtain the final contour lengths. AFM imaging was performed using a Multimode 8 AFM (Bruker, Billerica, MA, USA) at room temperature in the contact mode with the “E” scanner with 0.06 N/m cantilevers (SNL-10 probe, Bruker, Billerica, MA, USA). Images were recorded at a scan rate of 6 Hz and analyzed by Nanoscope Analysis (V1.80R2sr3, Bruker, Billerica, MA, USA).

### 2.6. AFM Sample Preparation and Imaging

The bilayer was made by forming two monolayers on mica using a Teflon well, as described previously [34]. In short, a lipid monolayer is formed at the air–buffer (buffer A, 10 mM sodium phosphate, pH 7) interface in small Teflon wells that have a side port. A monolayer-coated mica fragment (formed using a Langmuir trough) is then deposited onto this monolayer in the Teflon well to form the supported bilayer. After, the protein is injected through the side port to a final concentration of ~15 µg/mL. Following incubation of ~45 min, the sample was washed with buffer A and then taken to the AFM, always under solution. The first monolayer (directly facing mica) consisted of eggPC, and the second monolayer (facing the subtending solution) consisted of eggPC–cholesterol 1:1 (molar ratio). With the PFO^G57C/S190C^ sample, the sample was prepared in the same way, except that, prior to imaging, 5 mM DTT was added into the solution. AFM imaging was performed as described above. The histogram for the stoichiometry measurements was obtained from 32 images from five different samples.

### 2.7. Planar Bilayer Membrane Measurements

All electrophysiological measurements were performed with an BC-535 bilayer clamp amplifier (Warner Instruments, Holliston, MA, USA) paired with an LPF-8 8-pole Bessel Filter (Warner Instruments, Holliston, MA, USA). Planar membranes were made from decane solutions of 1-palmitoyl-2-oleoyl-*sn*-glycero-3-phosphocholine (POPC) and cholesterol mixed at a ratio of 45:55 mol%, and the total lipid concentration was 20 mg/mL. Membranes were formed across a 200 μm diameter hole in a Teflon cup, separating the apparatus into two chambers. Each chamber was filled with a 1 mL buffer (0.1 M NaCl, 10 mM HEPES, pH 7.0), and a pair of Ag/AgCl electrodes was used to record the transmembrane current with a holding potential of 50 mV. Afterwards, 6.5 μg PFO monomers incubated with 0.2% DDM were added to the cis chamber (1:1000 dilution) to form pores in the membrane. All channel signals were recorded with a low-pass filter frequency of 5 kHz and a sampling frequency of 25 kHz. The data were analyzed with Clampfit10.4.

## 3. Results

### 3.1. Multi-Stack Gel Electrophoresis Resolves a Range of Sizes of PFO Pore Complexes

Previous studies of either PFO prepore or pore complexes using SDS-AGE revealed a single, relatively uniformly sized band for the oligomers, with essentially undetectable levels of smaller-sized oligomers than this predominant oligomer [16,17,18,19,20,21,22]. This led to the suggestion that there is essentially only one dominant structure of either the fully formed prepore or the pore oligomer: namely, the complete ring of a specific size. However, subsequent microscopic studies clearly showed a range of sizes of the complexes, many of which are incomplete rings [23,24,25,26,27,28]. We reasoned that this apparent disagreement might be owing to a too-low resolving power of these gels to effectively separate differently sized complexes. Thus, to address this issue, we sought to examine PFO complexes with a continuous gradient gel that we expected would better separate such large, differently sized complexes than SDS-AGE. Pore complexes were examined by incubating PFO with cholesterol-containing lipid vesicles, followed by solubilization with a gel-loading buffer (that contains 0.5% lithium dodecyl sulfate (LDS)); then, the solubilized complexes were loaded into a 3–12% gradient polyacrylamide gel and electrophoresed. However, as shown in Figure 1A, we also observed only a single dominant band of a (detergent-resistant) higher-molecular-weight species, similar to previous results obtained with SDS-AGE [16].

We hypothesized that the resolving power of this gel might still not be sufficiently high enough to separate the differently sized complexes and thus systematically examined a number of gel-casting conditions (Supplementary Figure S1). Ultimately, we found that sequential stacks of different polyacrylamide density yielded the clearest separation of multiple well-defined bands (Figure 1B, Supplementary Figure S1, Section 2). We obtained similar results over a broad range of conditions, including different protein/vesicle incubation times (Figure 1B, Supplementary Figure S2), different gel densities (Supplementary Figure S1), and other sample conditions (Supplementary Figure S2). Thus, these results clearly show that gel electrophoretic-based methods can indeed reveal a range of sizes of the PFO pore complexes.

With up to many tens of subunits per pore complex, we expected that there would be likewise at least many tens of separate bands in the gel. However, instead, these gels generally showed only a few dominant bands. Though different gel compositions and conditions changed the presence of some bands or their relative dominance, the maximum number of bands that we routinely resolved was seven (Figure 1B, Supplementary Figure S2A). Hence, unexpectedly, these results indicate that the PFO pore complexes overall exhibit a strong preference for only a few different stoichiometries.

### 3.2. AFM Suggests That the Predominant Gel-Resolved PFO Pore Complexes Are Multiples of Six Subunits

Owing to the well-known contribution of both size and structure to the location of folded biomolecules in gels following electrophoresis [35], we sought to obtain reliable measurement of the size of the complexes by first isolating the complexes from each band and then imaging with AFM. To this end, we extracted the complexes from each gel-resolved band, deposited them on mica, and imaged them with AFM in solution. As shown in Figure 1C, overall, these images revealed both incomplete- and complete-ring complexes, with those complexes from the lower-weight bands exclusively arc-shaped and the higher-weight bands consisting mostly of complete rings. Thus, consistent with previous imaging-based observations [23,24,25,26,27,28,29,30], the gel electrophoresis assays also revealed the PFO pore complexes to exist as a range of differently sized complexes that are either incomplete or complete rings.

These AFM images were not of a sufficient resolution to resolve individual subunits within the complexes, likely owing to a poorer resolving capacity of AFM at sparse surface coverages of proteins on the substrate, a common issue with biological AFM [36]. Thus, we estimated the stoichiometry of these complexes based on their contour lengths using the known circumferential subunit length of the pore complexes (2.5 nm [24,31]). We found that the contour lengths within each band were narrowly defined, with sequential bands consisting predominantly of ~6, 12, 18, 24, 30, 36, and 42 subunits, respectively (Figure 1D). That is, overall, the PFO pore complexes are predominantly integral multiples of six subunits.

### 3.3. Confirmation of the Hexameric-Based Stoichiometry with High-Resolution AFM and Single-Channel Electrophysiology

**Figure 2:**
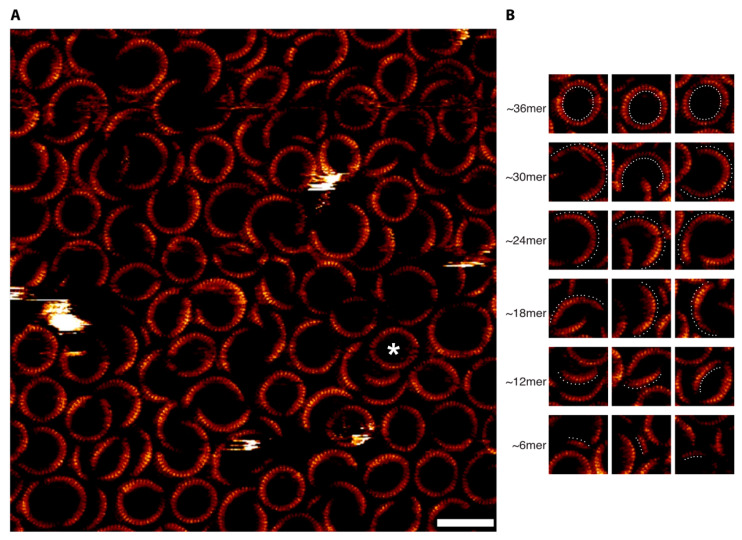
High-resolution AFM images of PFO pore complexes in supported lipid bilayers directly resolves subunit stoichiometry. (**A**) Large-scale image of the complexes in the supported bilayer. The asterisk indicates an example of a complex that appears to be formed by the apposition of two arcs of a similar curvature, as in previous studies [16,23,24,25,26,27,28]. Scale bar: 40 nm. (**B**) Isolated images showing the subunit stoichiometry indicated on the left, with white dots indicating the subunits in each complex.

**Figure 3:**
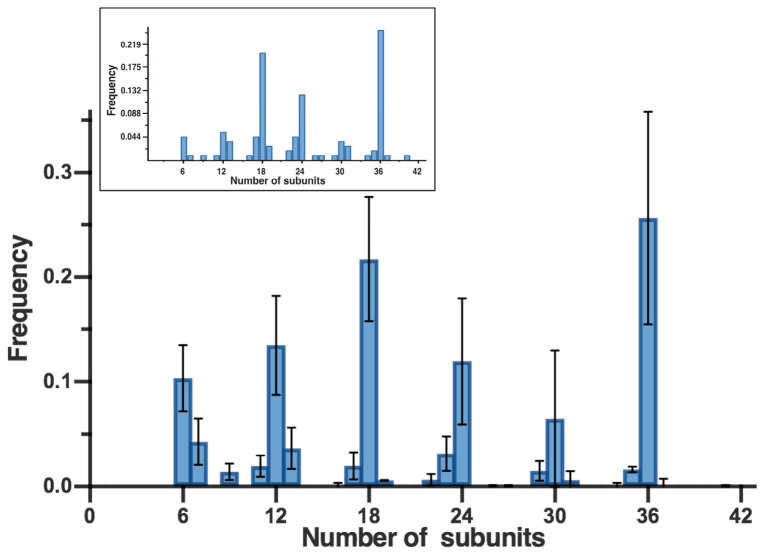
Distribution of subunit stoichiometries across all images. Shown is the average frequency of each subunit stoichiometry from individual images, together with the standard deviation. Overall, we analyzed 32 images from 5 different samples. Also shown in the inset is the frequencies of the stoichiometries of PFO pore complexes observed across all samples.

To verify this observation, we sought to directly image densely packed PFO pore complexes within supported lipid bilayers by in situ AFM [37,38,39,40]. As previously observed [24,37,39,40,41,42], such densely packed samples of membrane proteins can indeed yield AFM images with subunit-level resolution. Consistent with the AFM images of the gel-resolved bands (Figure 1C) and our previous observations [24], images of such complexes revealed both incomplete-ring and complete-ring complexes of a range of different sizes and curvatures with sufficiently high resolution to resolve individual subunits (Figure 2A). Directly counting the number of subunits from individual PFO complexes from such images indeed revealed a predominance of complexes that are integral multiples of six subunits, consistent with the gel results (Figure 2B and Figure 3).

We note that the aforementioned initial SDS-AGE study [16] also provided single-channel electrophysiological data that appeared to indicate a single predominant conductance of the complexes. Thus, we re-examined this experiment as well by studying single channels formed directly in cholesterol-containing planar lipid bilayers (Section 2). However, rather than a single predominant conductance, we observed a wide range of conductances (Supplementary Figure S3), consistent with the formation of a wide range of pore complex sizes observed with AFM and in the multi-stack gels.

### 3.4. Multi-Stack Gel Reveals Hexameric Stoichiometry in Prepore-Trapped PFO Complexes

Thus, altogether, these results show that PFO pore complexes are predominantly integral multiples of six subunits. Given that PFO is known to form pores by a prepore intermediate, we reasoned that insight into the role of hexamerization in the pore formation process would be provided by an examination of the sizes that PFO prepores form. For example, if PFO prepore complexes instead formed a range of sizes, in contrast to the pore complexes, this observation would suggest that association into stable hexameric sub-complexes may be a critical step specifically during the transition from the prepore to pore conformation.

**Figure 4:**
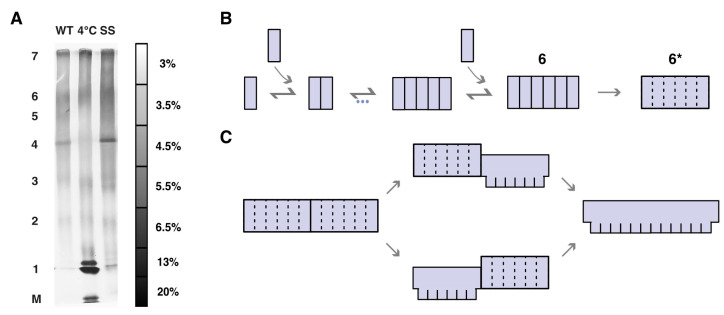
Multi-stack gel of prepore complexes provides support for a novel model of the pore-forming mechanism. (**A**) Prepore complexes (PFO^G57C/S190C^, labeled SS in the figure, and wild-type PFO at a lower temperature (4 °C)) resolve into the same few bands (maximally seven) that were observed with the pore complexes. The wild-type PFO lane to the left is the same lane to the right in Figure 1B to enable comparison. (**B**) Proposed model to explain the observations presented here, whereby upon forming hexamers, the complexes undergo a transition to a structure in which subunits now exhibit highly cooperative behavior (depicted by the dotted lines). (**C**) Proposed model in which the structural transition from prepore to pore occurs cooperatively within each hexamer, with a weaker coordination between neighboring hexamers (separated by solid line).

Thus, we also examined the sizes of two different prepore-trapped complexes with the multi-stack gel: a disulfide-locked mutant (PFO^G57C/S190C^) that was previously shown to assemble only into prepore complexes in the absence of dithiothreitol (DTT) [24,43] and complexes of wild-type PFO that assemble at a lower temperature (4 °C) [16] that only form prepores and not pores. As Figure 4A shows, prepore-trapped complexes formed via either of two different ways exhibit the same few bands as the pore complexes, also maximally seven. Thus, these results indicate that there is a striking preference for hexameric-based complexes in both the prepore and pore states.

### 3.5. Identification of a Mutant That Is Kinetically Trapped at a Hexameric State

Hence, overall, these results reveal a hierarchical subunit organization within both PFO prepore and pore complexes. Further, this suggests the presence of an important step upon hexamerization within the prepore state that persists to the pore state. In particular, we propose that monomers assemble on the membrane until forming hexamers (“6”), at which point they undergo a transition to a more cooperative (detergent-resistant [44]) hexameric prepore complex (“6*”) that is then able to convert to the pore state (Figure 4B).

**Figure 5:**
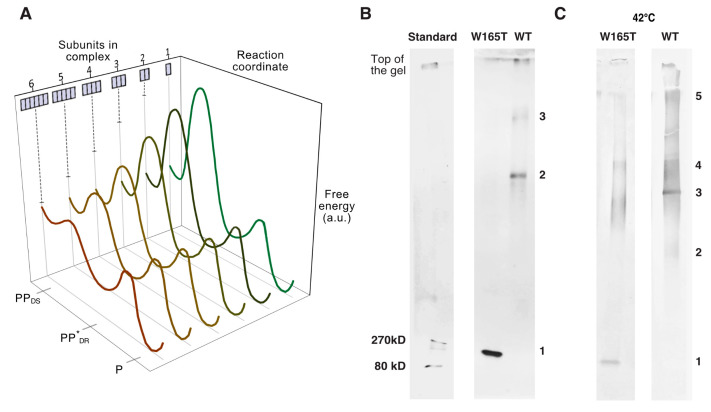
The PFO^W165T^ mutant is kinetically trapped at a hexameric state at room temperature. (**A**) Schematic diagram of the energy landscape proposed to underlie the prepore to pore transition for a monomer within the membrane associated complex. There is a barrier separating the (detergent-sensitive) prepore conformation from the insertion-competent (detergent-resistant) prepore conformation (*) that is reduced with the addition of each subunit to the complex. When there are 6 subunits, the barrier is small enough to be overcome by thermal fluctuations. From this *-state, the barrier to the pore conformation is likewise small enough to be overcome by thermal fluctuations. PP_DS_—detergent-sensitive prepore conformation; PP*_DR_—detergent-resistant prepore conformation; P—pore conformation. (**B**) Multi-stack gel of complexes of PFO^W165T^ formed on vesicles at room temperature shows only a single band, at the location of the wild-type hexamers. (**C**) A similar gel of the complexes of PFO^W165T^ formed on vesicles at 42 °C shows larger-sized complexes similar to wild-type PFO.

In energetic terms, this 6-to-6* transition can be understood as owing to an energy barrier separating the conformation of the subunit in sub-hexameric complexes from the conformation of the subunit in the 6*-complex, with the binding of additional subunits to the sub-hexameric complex (from dimer to trimer and so on) lowering the magnitude of this barrier (Figure 5A). In this scenario, only when the complex has become a hexamer is the barrier low enough to be overcome by thermal fluctuations. Accordingly, we expected that there should be mutations that substantially increase the magnitude of the barrier, prevent this 6-to-6* transition at room temperature, and thereby prevent pore formation (though not assembly). While a thorough examination of potential mutations that might produce this effect is beyond the scope of the present work, we noticed that prior published work indeed described a single-point mutation of tryptophan-165 to threonine that results in a mutant (PFO^W165T^) that assembles into large (linear) complexes but does not form pores at room temperature [45,46]. Thus, we examined PFO^W165T^ with the multi-stack gel and indeed found that at room temperature, this mutant assembles only up to a hexameric state but does not form larger-sized complexes (Figure 5B). Further, in agreement with the energetic scheme mentioned above, when incubated at higher temperatures (42 °C), PFO^W165T^ was indeed found to form larger-sized complexes of the same hexameric-based sizes as those that are formed by wild-type PFO (Figure 5C). Thus, together, these observations are consistent with the idea that this mutation produced a protein in which the 6-to-6* barrier is greater than in the wild type, so that at room temperature, the mutant was kinetically trapped at a hexameric state (but able to overcome this barrier at 42 °C), in agreement with our model.

## 4. Discussion

One of the major outstanding problems in our understanding of PFTs in general and CDCs in particular is how structural changes are coordinated between different subunits within a single complex during pore formation. With the CDCs, an early model based on SDS gel electrophoresis data suggested complete coordination among all subunits in the complex upon formation of a complete ring. However, extensive microscopic evidence indicated that pore complexes can also be incomplete rings, which thus led to the question of why the gel electrophoretic assay was only apparently detecting complete rings. In this work, we developed a novel gel electrophoretic assay that provides data to resolve this issue but, at the same time, also unexpectedly revealed a previously unknown structural hierarchy of these complexes that likely plays a significant role in the coordination of structural changes within the entire complex.

In particular, our multi-stack gel electrophoretic assay, for the first time, clearly separated multiple bands of the complexes, compared with the single band observed with SDS-AGE in previous studies or a continuous 3–12% native gradient PAGE. Subsequent AFM imaging of the complexes in each band revealed both complete and incomplete rings, consistent with previous imaging-based methods of these complexes [23,24,25,26,27,28]. Thus, this stepwise gel assay evidently enabled the finer separation of complexes of different stoichiometry from within a population of a mixture of differently sized complexes, which may also prove useful for studies of other large protein complexes.

However, we found that there were only seven predominant sizes of the PFO pore complexes, and that their stoichiometry differed by integral multiples of six subunits, a result confirmed by in situ AFM and electrophysiological data. Based on observations of complexes in the prepore state and the PFO^W165T^ mutant, we propose a model in which, upon hexamerization, there is a change in the nature of subunit–subunit interactions such that these interactions differ within the hexamers from those between the hexamers (Figure 4B and Figure 5A), which we suggest would lead to a likewise difference in the coordination of structural changes within/between the hexamers. Such hierarchical functioning of the complex may also be a property of other members of the MACPF/CDC family of toxins and also other PFTs.

Indeed, to our knowledge, such a hierarchical assembly in other CDCs has not been described before, although we note that it may be a feature of the bi-component β-PFTs, as the final pore complex of these PFTs consists of multiples of pairs of toxins [47,48]. Nonetheless, there are some published data that indicate that such a hexameric-based stoichiometry might be a feature of other CDC members. For example, the high-resolution pore structure of pneumolysin obtained by cryo-EM is a complex composed of 42 subunits [31]. Presumably, this was the predominant class size of these complexes. However, it is not clear what other sizes of the complexes, if any, were observed in this work, as no other data in this regard were published. In addition, in a previous AFM study of suilysin, the pore complexes appear to exhibit peaks in the size distribution of 18-mers and 24-mers [25]. However, these histograms are unfortunately not binned at the single-subunit-level resolution needed to fully clarify whether or not there were other sizes also preferred. Thus, we hope that, in the future, a re-investigation of previous unpublished data might provide clarification of the generality of the feature described in this work.

We note that, according to our model, the conversion to the pore conformation occurs simultaneously (cooperatively) within this hexamer, with neighboring hexamers transitioning either independently or with reduced cooperativity than within the hexamers (Figure 4C). As such, this description recalls the nested model of allostery of other large biological complexes [14], with stronger coordination within the hexamers than between them. With this, during pore formation, any single complex (larger than a hexamer) would be at least transiently composed of sub-complexes in the prepore state as well as those in the pore state (simultaneously). We note that this has indeed been recently observed with high-speed AFM imaging of another CDC member [49] and in our earlier study of a disulfide-trapped PFO mutant following reduction (Supplementary Figure S4).

We also note that there are two possible mechanisms by which complexes grow in size after attaining this cooperative hexameric prepore state: with monomers continuing to bind to this prepore or with exclusive interactions between cooperative hexamers (Supplementary Figure S5). Of note, the former mechanism affords the possibility of monomers binding to a complex that has already partially transitioned to the pore conformation, which could explain the continued growth of a pore complex observed in recent single-molecule fluorescence microscopy studies [29,30].

## 5. Conclusions

Based on the analysis of both gel electrophoretic data and high-resolution AFM images, we have shown, for the first time, that PFO pore complexes are predominantly multiples of six subunits, which we anticipate plays a critical role in coordinating structural changes between the subunits to the pore state. Thus, unlike what is commonly believed, the subunits within a complex are not all equivalent, with those within a hexamer interacting in a different way than those between hexamers. At the same time, these results re-focus attention to a major unresolved issue that the only-complete-rings model was purported to resolve: the trigger to pore formation, which indeed remains unanswered for many PFTs. In our model, this trigger occurs following hexamerization and predominantly at the level of this hexamer, but the nature of this trigger indeed remains unresolved. We anticipate that future studies of this hexamer should provide mechanistic insight into this critical question. Further, we anticipate that resolution of this problem will also shed light into the more general question of structural coordination mechanisms within nested protein architectures that underlie the functioning of higher-order biological assemblies more broadly.

## Data Availability

Data are contained within the article and Supplementary Materials.

## Supplementary Materials

The following supporting information can be downloaded at https://www.mdpi.com/article/10.3390/biom15030424/s1, Figure S1: Detection of different numbers of bands in gels of different compositions; Figure S2: Different sample conditions examined with the multi-stack gradient gels; Figure S3: Histogram of the distribution of channels formed by PFO in POPC–cholesterol planar bilayers from 11 separate planar membrane experiments; Figure S4: AFM images of PFO^G57C/S190C^ show the co-occurrence of prepore and pore sub-complexes within a common complex after a brief incubation with DTT, followed by fixation with 2% glutaraldehyde; Figure S5: Schematic diagram showing two mechanisms that reflect how complexes could possibly grow after achieving the cooperative hexameric prepore state (dotted line). And Original multi-stack gels.

## Abbreviations

The following abbreviations are used in this manuscript:

CDC	Cholesterol-dependent cytolysins
PFT	Pore-forming toxin
MACPF	Membrane attack complex/perforin-like protein
PFO	Perfringolysin O
SDS-AGE	Sodium dodecyl sulfate-agarose gel electrophoresis
AFM	Atomic force microscopy
Egg-PC	Egg L-α-phosphatidylcholine
PAGE	Polyacrylamide gel electrophoresis
POPC	1-Palmitoyl-2-oleoyl-sn-glycero-3-phosphocholine
LDS	Lithium dodecyl sulfate
